# Military sexual trauma-related posttraumatic stress disorder service-connection: Characteristics of claimants and award denial across gender, race, and compared to combat trauma

**DOI:** 10.1371/journal.pone.0280708

**Published:** 2024-01-11

**Authors:** Aliya R. Webermann, Mayumi O. Gianoli, Marc I. Rosen, Galina A. Portnoy, Tessa Runels, Anne C. Black

**Affiliations:** 1 VA Connecticut Healthcare System, West Haven, CT, United States of America; 2 Department of Psychiatry, Yale School of Medicine, New Haven, CT, United States of America; 3 Department of Psychiatry, University of Connecticut School of Medicine, Farmington, CT, United States of America; 4 Department of Internal Medicine, Yale School of Medicine, New Haven, CT, United States of America; Uniformed Services University of the Health Sciences, UNITED STATES

## Abstract

The current study characterizes a cohort of veteran claims filed with the Veterans Benefits Administration for posttraumatic stress disorder secondary to experiencing military sexual trauma, compares posttraumatic stress disorder service-connection award denial for military sexual trauma-related claims versus combat-related claims, and examines military sexual trauma -related award denial across gender and race. We conducted analyses on a retrospective national cohort of veteran claims submitted and rated between October 2017-May 2022, including 102,409 combat-related claims and 31,803 military sexual trauma-related claims. Descriptive statistics were calculated, logistic regressions assessed denial of service-connection across stressor type and demographics, and odds ratios were calculated as effect sizes. Military sexual trauma-related claims were submitted primarily by White women Army veterans, and had higher odds of being denied than combat claims (27.6% vs 18.2%). When controlling for age, race, and gender, men veterans had a 1.78 times higher odds of having military sexual trauma-related claims denied compared to women veterans (36.6% vs. 25.4%), and Black veterans had a 1.39 times higher odds of having military sexual trauma-related claims denied compared to White veterans (32.4% vs. 25.3%). Three-fourths of military sexual trauma-related claims were awarded in this cohort. However, there were disparities in awarding of claims for men and Black veterans, which suggest the possibility of systemic barriers for veterans from underserved backgrounds and/or veterans who may underreport military sexual trauma.

## Introduction

According to the U.S. Department of Defense, rates of military sexual trauma (MST; sexual harassment and sexual assault experienced during military service) have increased annually since 2018, yet only one-fifth of incidents are reported to military officials [1]. Service members and veterans of all genders, races, and backgrounds experience MST, though MST is substantially more likely to be endorsed among women compared to men [2–4]. Findings vary as to whether MST is more likely to be endorsed by White veterans compared to Black and other racial and ethnic minority veterans [2, 4], though a recent study found that Black and Latina women veterans were less likely than White women veterans to disclose MST as part of standardized Veterans Health Administration (VHA) MST screenings [5]. MST is strongly associated with multiple mental and physical health conditions, most notably posttraumatic stress disorder (PTSD), and veterans who experience MST report more severe PTSD symptoms compared to veterans who experience childhood abuse, non-MST sexual assault, and combat [2, 6]. Men often demonstrate a differential mental and physical sequalae following MST compared to women. They also appear to underreport MST during active duty and as veterans, which may be due to uniquely negative reactions to MST disclosure, a disrupted sense of masculinity, and discomfort in seeking MST care [7–9].

Veterans who develop or experience worsened mental health issues, including PTSD symptoms, following MST may file a claim with the Veterans Benefits Administration (VBA) within the Department of Veterans Affairs (VA) to receive tax-free monetary compensation and covered healthcare benefits [10]. As of 2014, approximately 95% of MST-related applications claimed PTSD [11]. There has been a marked increase in the number of MST-related PTSD claims filed with VBA in recent years; a 2018 report by the VA’s Office of the Inspector General (OIG) found that approximately 12,000 MST-related claims were filed with and completed by VBA annually in the preceding three years [12], while in fiscal year 2022, 28,354 MST-related claims were filed with and completed by VBA (C. Rawls, D. McDonough, personal communication, June 27, 2023).With approximately 118,000 total PTSD claims filed and completed by VBA in 2020 [13], MST-related claims comprise approximately one quarter of all PTSD claims filed with and completed by VBA.

The processing of MST-related PTSD claims is a multistep process that first involves having a Veterans Service Representative (VSR) review the veteran’s claim, military service records, military treatment records, and post-service treatment records for evidence that the MST occurred and is linked to the claimed symptoms. Given that the vast majority of MST goes unreported to military officials, VSRs typically use “markers,” i.e., indirect and circumstantial evidence of behavioral changes occurring during or around the time of the MST, such as requests for transfer, substance misuse or abuse, behavioral or occupational issues, or mental health complaints. Notably, evidentiary requirements for personal trauma claims, which includes MST as well as robbery, battery, stalking, and other events, are distinct from PTSD claims secondary to combat or fear of hostile military or terrorist activity, which do not require markers and for which a veteran’s military discharge papers and separation documents (i.e., DD Form 214) are sufficient to establish the occurrence of the stressor [10]. If VSRs find credible evidence of MST and/or markers, a compensation and pension (C&P) examination is ordered. C&P exams for PTSD are mental health evaluations conducted by doctoral-level licensed clinicians to determine if the veteran has a disability due to a diagnosable mental health condition related to military service. VBA adjudicators consult the C&P exam report with other records to render decisions abouts service-connection, which is calculated using the VA Schedule for Rating Disabilities [10].

Reports from governmental and non-profit organizations have brought attention to issues in the disability claims process for MST-related PTSD claims and potential disparities in awarding of MST-related PTSD claims relative to non-MST claims, such as for combat. From 2008–2012, the grant rate for MST-related PTSD claims was 16.5–29.6% less than for other PTSD claims, such as those related to combat [14]. Additionally, in 2018 and 2021, VA’s OIG reported that approximately half of denied MST claims were not properly processed under VBA procedures, including not ordering a C&P exam when one was warranted, not gathering evidence adequately, and using insufficient medical opinions to decide claims [12, 15]. As of 2018, according to the Center for a New American Security, awarding of PTSD claims for MST-related claims and non-MST-related claims was nearly identical at around 54–56% each [16], and per VA, the percentage of awarded MST-related PTSD claims has increased from 35.6% in 2011 to 72% in 2021 [17]. Although women veterans are less likely overall to be awarded PTSD claims than men veterans [18], these aforementioned reports have noted that men are less likely to be awarded MST-related PTSD disability than women. As of 2018, the MST-related award rate for men was 44.7% compared to 57.7% for women [16]. However, no empirical research to date has examined disparities in MST-related PTSD claim awarding across gender.

Although not focused on MST claims specifically, prior reports [19] and empirical studies [18, 20] have documented a longstanding pattern of racial disparities in the awarding of PTSD claims, resulting in more claim denials and lower ratings for Black veterans relative to White and other non-Black veterans, especially when psychometrically validated diagnostic measures are not used [21]. The topic of racial disparities in awarding of PTSD claims has been a focus of substantial media and VA interest since 2022, when a Black veteran filed a lawsuit in federal court alleging racial disparities in VA’s awarding of disability claims and other benefits [22]. The lawsuit presented VA data which found that between 2001–2020, the VA was 21.9% more likely to reject claim applications from Black veterans than White veterans [22].

Prior work has reported disparities in awarding of PTSD claims across gender and race, MST claims across gender, and for MST claims compared to non-MST claims, demonstrating a critical need for additional empirical analysis to identify who might be underserved in accessing disability benefits earned through service. Receipt of PTSD benefits is associated with subsequent stable or increased mental healthcare use among veterans [23], reinforcing the necessity of accurate and fair service-connection decisions. To gain further insight into patterns of awarding of MST-related PTSD claims, we used a national veteran sample to address the following study aims: 1) Compare PTSD service-connection award decisions for MST versus combat claims; 2) characterize a cohort of veterans who filed for MST-related PTSD service connections within a 5-year time period; and 3) examine differences in MST-related PTSD service-connection award decisions across gender and race.

## Method

### Study design and sample

The present study is a secondary analysis of a retrospective cohort of 125,831 veterans who filed 134,207 initial (new) and/or supplemental (review or appeal) claims with VBA for PTSD service-connected disability benefits, and had claims completed and rated by VBA between October 1, 2017 and May 19, 2022. This timeframe of data was used as VBA started specifically identifying MST claims as a subset of personal trauma-related PTSD claims in their data in fiscal year 2018 (P. Kamath, P. Jong, personal communication, May 19, 2022). Data were requested on May 19, 2022 through a written request to VBA’s Office of Performance Analysis and Integrity, which provides VBA claim data by request for use only by VA users and within the VA firewall. We requested data for all veterans who filed PTSD claims during this period. This data set has not been used for any empirical publications to date. The data included 31,803 MST-related PTSD claims (92.2% initial, or *n* = 29,317) and 102,409 combat-related PTSD claims (92.1% initial, or *n* = 94,328). While most veterans (*n* = 118,357) filed one claim, veterans could have filed multiple claims for PTSD, and the number of times that veterans filed PTSD claims ranged from 1–10 (*M* = 1.14, *SD* = 0.44). Veterans could file PTSD claims for both MST and combat (*n* = 1,364), or could file a supplemental claim if their initial claim was denied, if requesting a service-connection increase, or if the claim was undergoing VBA review (Table 1). PTSD claims not related to MST or combat were not included in this analysis. The present study was approved by the VA Connecticut Healthcare System Institutional Review Board Research and Development Committee in December 2022. The project was determined to be exempt from Human Subjects Subcommittee review as secondary research using data collected by a government agency (i.e., VBA) for which informed and/or written consent is not required.

**Table 1:** Number of PTSD claims filed per veteran (*N* = 134,207).

1	2	3	4	5	6	7	8	9	10
118,362	13,498	1,716	508	45	36	21	16	0	10

MST claimants were primarily women (79.7%), non-Hispanic White (55.5%), Army veterans (45.4%), non-commissioned officers (65%), did not serve post-9/11 (62.6%) and averaged 35.77 years old (*SD* = 8.89, range = 19–79; Table 2). Combat claimants were primarily men (92.3%), non-Hispanic White (69%), Army veterans (68.3%), non-commissioned officers (80.1%), served post-9/11 (72.4%), and averaged 41.08 years old (*SD* = 8.82, range = 19–87; Table 3). For both MST and combat-related claims, service-connection denial rates were highest among Black veterans, Navy veterans, and formerly enlisted personnel (Tables 2 and 3). Men were more likely than women to be denied MST-related claims, but less likely than women to be denied combat-related claims (Tables 2 and 3).

**Table 2:** Characteristics of MST-related PTSD claims (*N* = 31,803).

	Claim denied n (% within category*)	Total number of claims filed (% of MST claim N**)
**Gender**		
Women	6,399 (25.4%)	25,193 (79.7%)
Men	2,343 (36.6%)	6,397 (20.3%)
**Race/ethnicity**		
White, not of Hispanic origin	4,438 (25.3%)	17,568 (55.5%)
Black, not of Hispanic origin	3,176 (32.4%)	9,801 (31.0%)
Asian or Pacific Islander	370 (26.6%)	1,389 (4.4%)
American Indian/Alaskan Native	172 (26.6%)	646 (2.0%)
Unknown	582 (26.1%)	2,227 (7.0%)
Hispanic origin	38 (23.5%)	162 (0.5%)
**Age *M*, *Med* (SD, range)**	37.54, 37.00 (8.84)	35.77, 35.00 (8.89, 19–79)
**Post-9/11**	3,316 (27.9%)	11,903 (37.4%)
**Highest rank**		
Non-Commissioned Officers	5,376 (26.6%)	20,241 (65.0%)
Enlisted Personnel	2,873 (31.4%)	9,163 (29.4%)
Warrant Officers	25 (17%)	147 (0.5%)
Commissioned Officers	327 (20.5%)	1,597 (5.1%)
**Branch**		
Army	4,080 (28.3%)	14,424 (45.4%)
Navy	2,333 (29.1%)	8,018 (25.2%)
Air Force	1,433 (25.2%)	5,696 (17.9%)
Marine Corps	845 (26.2%)	3,228 (10.2%)
Coast Guard	89 (21.5%)	414 (1.3%)
Other (Space Force, Commissioned Corps, etc.)	8 (36.4%)	22 (0.1%)

*Percentages are of claims denied across all claims filed within a characteristic category (e.g., 25,193 women filed MST-related claims, and 6,399 of these claims, or 25.4%, were denied).

**All *n*’s do not = 31,803 due to missing data. Percentages are of total number of claims filed within a characteristic category (e.g., 14,424 of the 31,803 MST-related claims, or 45.4%, were filed by Army veterans).

**Table 3:** Characteristics of combat-related PTSD claims (*N* = 102,409).

	Claim denied n (% within category*)	Total number of claims filed (% of combat claim N**)
**Gender**		
Women	1,588 (20.2%)	7,843 (7.7%)
Men	16,994 (18.0%)	94,263 (92.3%)
**Race/ethnicity**		
White, not of Hispanic origin	11,927 (17.0%)	70,197 (69.0%)
Black, not of Hispanic origin	4,643 (23.1%)	20,067 (19.7%)
Asian or Pacific Islander	977 (16.0%)	6,105 (6.0%)
American Indian/Alaskan Native	216 (18.1%)	1,193 (1.2%)
Unknown	775 (18.3%)	4243 (4.2%)
Hispanic origin	214 (12.6%)	1696 (1.7%)
**Age *M*, *Med* (SD, range)**	40.92, 39.00 (9.54)	41.08, 40.00 (8.82)
**Post-9/11**	13,968 (18.8%)	74,132 (72.4%)
**Highest rank**		
Non-Commissioned Officers	14,084 (17.7%)	79,474 (80.1%)
Enlisted Personnel	2,664 (28.6%)	9,324 (9.4%)
Warrant Officers	218 (10%)	2,174 (2.2%)
Commissioned Officers	1,109 (13.5%)	8,225 (8.3%)
**Branch**		
Army	11,629 (16.6%)	69,985 (68.3%)
Navy	1,896 (27.6%)	6,882 (6.7%)
Air Force	2,134 (22.2%)	9,616 (9.4%)
Marine Corps	2,948 (18.7%)	15,741 (15.4%)
Coast Guard	43 (24.4%)	176 (0.2%)
Other (Space Force, Commissioned Corps, etc.)	0 (0%)	9 (0.009%)

*Percentages are of claims denied across all claims filed within a characteristic category (e.g., 94,258 men filed combat-related claims, and 16,994 of these claims, or 18%, were denied).

**All *n*’s do not = 102,409 due to missing data. Percentages are of total number of claims filed within a characteristic category (e.g., 94,258 of the 102,409-related claims, or 92%, were filed by men).

### Study variables and analyses

The data set from VBA included veteran age, gender, race, ethnicity, period of service, military branch, and rank (see Tables 2 and 3), and claim information including: service-connection award decision (i.e., awarded or denied); explanation for service-connection award decision (e.g., incurred/caused by service); basis for PTSD service-connection (i.e., MST or combat); percent PTSD disability rating from 0–100%; and relevant claim dates, including date of VBA claim receipt, date of claim authorization, and date of rating decision. The majority of study variables were categorical, including all claim information except for disability rating (continuous variable from 0–100%), and identity and military service characteristics except for age. The primary outcome was service-connection award decision, a dichotomous variable (awarded or denied).

Using the data set of combat-related and MST-related PTSD claims, we used binary logistic regression analysis to model the probability of initial PTSD service-connection award denial as a function of gender (binary gender based on birth sex, i.e., male or female), age, race (White, Black, Asian/Pacific Islander, American Indian/Alaskan Native, or unknown), and stressor type (i.e., combat or MST). All predictors were entered simultaneously. Looking specifically at MST claims, a second binary logistic regression model estimated the probability of MST service-connection award denial by gender, age, and race, entered simultaneously. The data met assumptions for logistic regression, including independent observations, as we only used initial claims and excluded supplemental claims from logistic regression analyses (VIF = 1.021–2.076).

## Results

### Descriptive information on MST and combat-related PTSD claims

Of the 31,803 MST claims submitted and rated, 72.4% (*n* = 23,015) were awarded and 27.6% (*n* = 8,788) denied. Comparatively, of 102,409 combat claims submitted and rated, 81.8% (*n* = 83,759) were awarded and 18.2% (*n* = 18,650) denied. The average MST-related PTSD service-connection award percentage was 60.35% (*SD* = 17.66), while the average combat-related PTSD service-connection rating was 55.27% (*SD* = 17.94). MST claim processing time was on average 4.78 months (*SD* = 4.41, range = 0–39 months) compared to 3.62 months (*SD* = 3.74, range = 0–40 months) for combat claims. The primary reasons for PTSD claim denial by VBA for both MST and combat claims were no diagnosis (i.e., does not meet established diagnostic criteria) and not incurred/caused by service (i.e., meets established diagnostic criteria but not related to claimed stressor(s); Table 4). A small number of MST-related (2.4%) and combat-related (0.8%) claims were denied for not being aggravated by service (i.e., previously met established diagnosed criteria which were not substantially aggravated by claimed stressor(s)) and other reasons (e.g., claimed stressor(s) did not occur during military service).

**Table 4:** Reasons for MST and combat-related PTSD claims denial.

	MST service-connection denied (*n* = 8,788)	Combat service-connection denied (*n* = 18,650)
**No Diagnosis**	4,647 (52.9%)	13,226 (71.5%)
**Not Incurred/Caused by Service**	3,923 (44.6%)	5,170 (27.7%)
**Not Aggravated by Service**	129 (1.5%)	29 (0.2%)
**Not Established by Presumption**	57 (0.6%)	101 (0.5%)
**Not in Line of Duty**	19 (0.2%)	5 (0.01%)
**Constitutional/Developmental Abnormality**	9 (0.1%)	8 (0.04%)
**Not Secondary**	3 (0.01%)	5 (0.03%)
**Not in Country**	1 (0.01%)	6 (0.03%)

### Differences in awarding for MST vs. combat-related PTSD claims

Table 5 shows the logistic regression modeling results with factors predicting denial of an initial PTSD claim (*n* = 122,130). Controlling for gender, age, and race, MST-related initial PTSD claimants had 2.05 times higher odds of being denied (27.6% denied) compared to combat-related initial PTSD claimants (18.2% denied), χ^2^ (7) = 1801.78, *p* < .001 [95% CI = 1.95–2.14]. Additionally, Black claimants (*p* < .001 [95% CI = 1.41–1.51]) and American Indian/Alaskan Native claimants (*p* < .001 [95% CI = 1.41–1.51]) were more likely to have their initial PTSD claims denied relative to White claimants. Age was also a significant predictor of claim denial in the model but the odds ratio does not appear to demonstrate a clinically meaningful difference in outcomes across age (*p* < .001 [95% CI = 1.00–1.01].

**Table 5:** Predictors of PTSD service-connection claim denial.

Variable	Odds ratio	95% CI	B	S.E.	Wald	*p* value
**Gender (reference: women)**	1.31	1.25–1.37	.27	.02	134.51	< .001
**Age**	1.01	1.00–1.01	.01	.00	52.80	< .001
**Race (reference: White)**						
**Black**	1.46	1.41–1.51	.38	.02	492.29	< .001
**Asian/Pacific Islander**	.96	.90–1.02	-.04	.03	1.52	.22
**American Indian/Alaskan Native**	1.14	1.00–1.14	.13	.07	4.43	.04
**Unknown**	1.07	1.00–1.14	.07	.03	3.82	.05
**Claim type (reference: combat)**	2.05	1.95–2.14	.72	.02	943.37	< .001

### Differences in awarding for MST claims across gender and race

Table 6 shows the logistic regression modeling results with factors predicting denial of an initial MST-related PTSD claim (*n* = 29,317). Controlling for age and race, men claimants had 1.78 times higher odds of MST service-connection denial (36.6% denied) compared to women claimants (25.4% denied), χ^2^ (6) = 862.60, *p* < .001 [95% CI = 1.67–1.89]. Black claimants had a 1.39 times higher odds of MST service-connection denial (32.4% denied) compared to White claimants (25.3% denied), χ^2^ (6) = 918.01, *p* < .001 [95% CI = 1.31–1.47]. Age was also a significant predictor of claim denial in the model but the odds ratio did not indicate a meaningful difference in outcomes across age (*p* < .001 [95% CI = 1.02–1.03].

**Table 6:** Predictors of MST service-connection claim denial.

Variable	Odds ratio	95% CI	B	S.E.	Wald	*p* value
**Gender (reference: women)**	1.78	1.67–1.89	.57	.03	333.21	< .001
**Age**	1.03	1.02–1.03	.03	.001	395.49	< .001
**Race (reference: White)**						
**Black**	1.39	1.31–1.47	.33	.03	122.57	< .001
**Asian/Pacific Islander**	1.08	.95–1.23	.08	.07	1.39	.238
**American Indian/Alaskan Native**	1.14	.94–1.37	.13	.10	1.79	.181
**Unknown**	1.09	.98–1.21	.08	.05	2.33	.127

## Discussion

The current study used a retrospective cohort of 125,831 veterans filing 134,207 initial or supplemental claims with VBA for PTSD service-connected disability benefits related to MST and/or combat-related traumatic stressors toward three research aims: 1) Compare PTSD service-connection award decisions for MST versus combat-related PTSD claims; 2) Characterize a cohort of veterans who filed for MST-related or combat-related PTSD service connections within a 5-year time period; and 3) Examine differences in MST-related PTSD service-connection award decisions across gender and race.

We found that while three-fourths of MST-related claims (72.4%) were awarded in a 5-year period, MST-related PTSD claims were significantly more likely to be denied (27.6% denied) compared to combat-related PTSD claims (18.2% denied). This disparity in claim outcomes in the current study is less than that reported in past research [14], but is concerning nonetheless. One consideration is that there are different evidentiary requirements for personal trauma claims such as MST, which require evidence of event occurrence (i.e., through a report to military officials, service treatment records for treatment of MST-related medical or psychiatric concerns, “markers,” or both), while for combat-related claims, discharge papers and separation documents are sufficient to establish the occurrence of the stressor [10, 12, 16]. As such, MST claims require more evidence than combat claims. Most MST is not reported during military service [1], and thus this potentially important proof of MST is not available to buttress the claim. Supporting this explanation is the finding that 44.6% of MST claims denials in the current sample were due to insufficient evidence, whereas only 27.6% of combat PTSD claims were denied for this reason.

In comparing characteristics between veterans who filed PTSD claims for MST versus combat, consistent with prior reporting, we found MST claims were primarily filed by women (79.9%) while combat-related claims were primarily filed by men (92.3%), and both types of claims were primarily filed by non-Hispanic White Army veterans. This speaks to the potential need for additional outreach to encourage filing of PTSD claims among racial minority veterans, and particularly among racial minority women veterans, as the population of women veterans is more racially diverse [24] than the general veteran population.

Lastly, we found that men veterans, as well as Black veterans, were more likely to be denied MST-related service-connection compared to women veterans and White veterans, respectively. This is unsurprising given prior work documenting perceived and experienced bias among men who disclose and seek treatment for MST [7–9], men applying for MST-service connection awards [16], and Black veterans applying for PTSD service-connection awards [18–20]. The processing of MST-related claims has been critiqued for procedural issues leading to improper claim denials [12, 15]. In recent years, the processing of MST claims has been revised to address these issues by having a more flexible evidentiary standard via the use of “markers,” limiting the processing of MST-related claims to specially trained personnel at only select VBA regional offices, and having MST claims undergo an additional level of review [12, 15].

Scholars have noted how the centering of White women within narratives around sexual assault, including military sexual assault, silences and deprioritizes sexual assault against men and Black individuals [25]. Further, Black women’s sexual victimization is often dismissed through harmful racialized tropes around an assumed hypersexuality [25]. Within this context and documented underreporting of MST by men [7–9] and Black veterans [4, 5], it is also possible these individuals are less likely to file a PTSD claim following an experience of MST. While reporting MST during service or to a VHA provider is not a requirement for an awarded MST-related disability claim, these reports and disclosures can serve as direct or indirect/circumstantial evidence to build a successful MST-related claim [10].

### Limitations and future research

This study was strengthened through using a national sample of PTSD claims for MST and combat filed, completed, and rated between October 2017-May 2022. There may have been historical and cultural factors unique to this timeframe, such as the #MeToo movement which gained substantial prominence in 2017, that uniquely impacted the filing, processing, and awarding of MST-related claims. The study sample included a substantially larger number of combat-related claims (102,409) than MST-related PTSD claims (31,803), though this is expected given that MST goes largely unreported within the military and VA [1, 4, 5] and MST claims have greater evidentiary requirements than combat-related PTSD claims [10].

Our study was focused on describing military and demographic characteristics of veterans filing PTSD claims for MST and combat exposure and predictors of PTSD and MST service-connection award denial, and thus excluded other potentially important covariates of PTSD service-connection awarding including PTSD symptom severity, comorbid mental and physical health issues and/or diagnoses (e.g., depression, substance use disorders), and locations that might impact claims decisions (i.e., VBA regional office where claim was filed, VHA setting where veteran completed their C&P exam, VHA where veteran sought care if applicable) [26, 27]. Furthermore, military and demographic variables were limited to administrative data routinely collected and reported by VBA and VHA. Without these other data, we do not know to what extent claim denials were uniquely explained by the factors we identified (type of claim, race, gender) or whether the factors we identified were collinear with other important predictors.

We utilized binary gender (i.e., male or female) rather than self-identified gender identity, and did not include sexual orientation, as VA did not routinely collect sexual orientation and gender identity data prior to 2022 [28, 29]. Prior work has found that sexual and gender minority veterans endorse higher rates of MST and associated PTSD symptoms compared to heterosexual and cisgender veterans [30], speaking to the importance of examining MST-related disability claims across sexual orientation and gender identity. An intersectional approach to conceptualizing MST, including the filing, processing, and awarding of MST-related claims, is warranted, wherein race, gender, age, sexuality, and other variables are considered in tandem rather than separately [25]. Lastly, future research should include interviews with veterans who file MST-related claims, which is crucial to better identifying areas of improvement in the processing of MST-related claims.

### Practice and policy implications

The process of filing, adjudicating, evaluating, and rating of MST-related PTSD claims is distinct from other PTSD and mental health claims, and has substantial impacts on the well-being and VHA care utilization decisions of the thousands of veterans entering this process annually [10, 16, 31]. Despite disability claims constituting the majority of VA’s annual budget [32], there is limited empirical data publicly available on MST-related claims processes and awards outcomes, but available data indicates the number of MST-related claims filed and awarded has increased in recent decades. While this is an encouraging indication of greater public acknowledgement of MST and its linkages to PTSD, there is not one singular “correct” rate of PTSD claim denial, for MST or combat. The various interested parties in the claims process–staff involved in processing MST-related claims, veterans filing claims, and policy-makers developing and overseeing VA policy and practice around MST-related claims–likely have differing perspectives on the appropriate number of claims to award or deny as well as the process in which awards are determined.

The association between race, gender and denial of MST claims may be due to a differently experienced process across race and gender among veterans who file MST-related claims. Interviews with VA staff who conduct C&P exams for MST describe unique challenges for men and racial minority veterans who file MST-related disability claims due to stigma, VA staff bias, and less documented evidence vis-à-vis markers [31]. These factors might influence how the evidence supporting claims is found and assembled, possibly in a way that makes claim denial more likely among men veterans and Black veterans. Different claims denial rates by race and gender might also be partially explained by differences in who files claims. However, there is no evidence to support men or Black veterans having a lower threshold for filing claims. It would be surprising if individuals who are less likely to report MST experiences in VA and military settings, such as Black veterans and men [4–9], would be more likely to file for MST-related disability compensation given the additional evidentiary requirements for MST-related PTSD claims. It is also unknown how many veterans potentially eligible for MST-related benefits do not file claims and thus are not considered in our analyses. There are veterans who would have filed MST-related claims but are ineligible for VBA disability benefits (for MST-related PTSD or otherwise) due to an other than honorable discharge, which some service members have previously indicated occurred to them as part of retaliation by their supervisors and other unit members for reporting MST [33].

Following a recent lawsuit alleging racial disparities in VA’s awarding of disability claims negatively impacting Black veterans [22], in June 2023, VBA formed an Equity Assurance Office, led by the VBA Deputy Executive Director for Policy, Procedures and Interagency Collaborations in Compensation Service [34, 35]. According to VA, the team is tasked with “making sure that we provide every Veteran with the world-class care and benefits they deserve–no matter their age, race, ethnicity, gender, religion, disability, or sexual identity. The team’s first order of business will be identifying any disparities in VA health care and benefits and eliminating them” [35]. It is unclear to what extent this team will focus on disparities within MST claims.

## Conclusion

Using a national cohort of 134,207 veteran PTSD disability claims filed and rated between 2017–2022, the present study compared PTSD claim denials for MST versus combat-related claims, described demographic and military characteristics of veterans who filed MST and combat claims, and examined differences in denial of MST-related PTSD claims across gender and race. We found that while three-fourths of MST claims were awarded, MST claims were two times more likely to be denied relative to combat claims, and men veterans as well as Black veterans were more likely to have their MST claims denied relative to women veterans and White veterans, respectively. More research is necessary to identify the root causes of gender and racial disparities in awarding of MST claims as well as disparities in awarding of MST-related PTSD claims relative to combat-related PTSD claims. Additionally, future research should examine the association between other potentially important covariates and denial of PTSD claims, and utilize an intersectional approach when assessing claim awarding across identity factors.
